# A Theoretical Basis for Entropy-Scaling Effects in Human Mobility Patterns

**DOI:** 10.1371/journal.pone.0161630

**Published:** 2016-08-29

**Authors:** Nathaniel D. Osgood, Tuhin Paul, Kevin G. Stanley, Weicheng Qian

**Affiliations:** 1 Dept. of Computer Science, University of Saskatchewan, Saskatoon, SK, Canada; 2 Dept. of Community Health and Epidemiology, University of Saskatchewan, Saskatoon, SK, Canada; Universidad Rey Juan Carlos, SPAIN

## Abstract

Characterizing how people move through space has been an important component of many disciplines. With the advent of automated data collection through GPS and other location sensing systems, researchers have the opportunity to examine human mobility at spatio-temporal resolution heretofore impossible. However, the copious and complex data collected through these logging systems can be difficult for humans to fully exploit, leading many researchers to propose novel metrics for encapsulating movement patterns in succinct and useful ways. A particularly salient proposed metric is the mobility entropy rate of the string representing the sequence of locations visited by an individual. However, mobility entropy rate is not scale invariant: entropy rate calculations based on measurements of the same trajectory at varying spatial or temporal granularity do not yield the same value, limiting the utility of mobility entropy rate as a metric by confounding inter-experimental comparisons. In this paper, we derive a scaling relationship for mobility entropy rate of non-repeating straight line paths from the definition of Lempel-Ziv compression. We show that the resulting formulation predicts the scaling behavior of simulated mobility traces, and provides an upper bound on mobility entropy rate under certain assumptions. We further show that this formulation has a maximum value for a particular sampling rate, implying that optimal sampling rates for particular movement patterns exist.

## Introduction

The importance of understanding how humans move through, consume and interact with the space they inhabit is a central tenet of geography, urban planning, architecture, and many other social sciences. Being able to concisely represent the quality of human movement through space allows practitioners in these disciplines to design better cities, buildings, and policies. Traditionally, human motion was studied using the pen-and-paper tools of the anthropologist, including retrospective surveys, direct observation, ethnography, or self-report through interviews or diaries. While these techniques have provided remarkable insight into human mobility, particularly into its cognitive aspects, they are limited in spatio-temporal resolution, and are prone to observer or reporter bias, and can be time consuming. Technological advances in localization have opened new opportunities for analyzing human mobility [1] [2].

Electronically mediated population tracking is a practical alternative to traditional pen and paper techniques. Inexpensive loggers or smartphone apps can use the Global Positioning System (GPS) to record trajectories through space [3] [4] [5]. While GPS-based systems provide exceptional positioning quality and coverage when outdoors, they can be unreliable in institutional buildings or in terrain where sky views are blocked. GPS-based data acquisition can also be more cumbersome as participants have to be recruited, potentially outfitted with appropriate equipment and debriefed. An alternate approach is to mine cell tower or WiFi router contact traces through time to generate trajectories by representing the locations of the device and, therefore, the person, as the locations of the towers or routers to which the device is connected (e.g., [6]). In proximity-based representations, space is implicitly represented as a sequence of polygons, derived from the Voronoi diagram of the beacons. While these representations can be easier to obtain, as cell or router contact records are often maintained by telecommunication companies or institutions, they are also often characterized by a heterogeneous spatial decomposition (based on the Voronoi diagram structure) and intermittent sampling, as records are often only generated for active connections (calls, texts, or data transmission).

These technologically mediated localization systems provide much higher spatial and temporal fidelity than traditional methods, are less prone to bias, but are divorced from the cognitive processes underlying the decision making. The additional spatio-temporal resolution can be a double edged sword, as traditional statistical analysis techniques suitable for analyzing survey responses are no longer sufficient for characterizing such data. To address the overabundance and complexity of the data, researchers have looked at visualization methods or statistical metrics to represent the important components of the data more concisely. Binned or aggregate statistical representations are popular. Heatmaps, visualizations of the two dimensional frequencies of parameters of interest, are a standard method of aggregating location over time and space (e.g., [7, 8]). Space is typically binned at a specific resolution, then location data is accumulated for each bin. Aggregate distributions of secondary measures can also be useful to summarize high fidelity data. Aggregate measures such as visit frequency, trip duration, trip length, and radius of gyration have been previously reported in the literature [9–12]. In all of these representations, spatio-temporal variation is marginalized over some variable, destroying important information about the structure of the variability. However, several researchers have observed simple and reproducible patterns and a high degree of spatial and temporal regularity in visited locations of humans [13–16].

In their seminal paper, Song et al. [9] proposed the entropy rate of a mobility pattern as a metric of variability or predictability in human behaviour. By discretizing the world, and providing a label to each discretized location, a trajectory through space could be converted into a string of location labels or symbols. As a string, this representation could be summarized by the entropy rate, which is closely related to the compressibility of the string. People with a great deal of regularity in their schedules would be represented by a lower entropy rate than people whose spatio-temporal habits were less predictable. This metric had the advantage of providing a measure of the regularity of spatio-temporal habits of a population as a single number. Song et al.’s original work has been extended to other aspects of human behavior, including social contact and activity in both complete and moving average implementations [17] [18] [19].

According to Shannon’s original definition, entropy is calculated directly from a random variable or distribution [20] [21]. Entropy could be calculated for aggregated distributions such as trip length or dwell time, but that representation does not capture the empirical entropy rate for the trajectory string. To approximate entropy rate empirically, lossless compression algorithms are generally employed [22]. In particular, the Lempel-Ziv 78 (LZ) algorithm has been shown to provide asymptotic estimates for the entropy rate of a string as the length of the string goes to infinity [9] [22] [23]. Following the example established in Song et al.’s original paper, researchers estimate the entropy rate of a mobility string through LZ compression, although shortcomings with this approach have been noted [24].

Employing the methodology originally proposed by Song et al., it is possible to use LZ compression to approximate the entropy rate of a person’s trajectory. However, the entropy rate calculated for this path is not universal, as it depends on the spatial and temporal resolution with which the path is sampled. That is, the resolution of binning and the regularity and rate of sampling impact the entropy rate calculated from the LZ compression technique [24] [25] [26]. Meaningful comparisons of entropy rates between different people or populations can only occur if those rates were calculated from strings with identical spatial and temporal resolution. This implies that meaningful comparison of mobility entropy across experiments is not possible in general, as the experimental protocol changes. It further implies that comparing different individuals in the same dataset could be problematic if there is heterogeneity in the geographic bin size or sampling rate; for example, in a study comparing the mobility of rural and urban populations through cell phone records, where the rural Voronoi cells were systemically and significantly larger than their urban counterparts.

Because mobility entropy rate is a useful metric, some researchers have studied or proposed empirical methods of describing variations in spatio-temporal scale [24] [25] [26]. However, empirical models can be difficult to generalize, as specific models may be tightly tied to the datasets from which they were derived. In this paper, we provide a theoretical derivation of a scaling law for mobility entropy rate calculated through Lempel-Ziv compression. This derivation is theoretically valid for non-overlapping trajectories which can be represented as a series of line segments navigated at constant velocity over a regular four-connected grid. This scaling model shows excellent agreement with simulated trajectories, even when those trajectories violate assumptions underlying the derivation. Analysis of the mathematical properties of the model yields several key findings. First, variation with spatio-temporal scale is an inevitable consequence of the LZ approximation. Second, mobility entropy rate at any spatio-temporal scale can be represented by four parameters: the length of the trajectory, the velocity of each segment and the spatial and temporal scales. Third, the model has a unique maxima with respect to the temporal sampling rate, implying that there is a natural sampling rate for a given trajectory which maximally captures the information it encodes. Finally, the performance of this model indicates it might be possible to express mobility entropy rates measured with different experimental configurations at common resolutions, allowing comparison between disparate populations and experiments, allowing mobility entropy rate to be employed to its full potential as a metric.

## Analysis

### Problem Structure

Our derivation relies upon the performance of Lempel-Ziv (LZ) compression in approximating the entropy rate of mobility, the most common method for estimating entropy rate based on the seminal work of Song et al. [9]. As many other researchers have noted [22, 23], this approximation makes strong assumptions about the behavior of the string, notably that it represents a stationary ergodic process, and is sufficient long for the algorithm to converge. While these assumptions may be violated in practice, the approximation is widely used in the literature. Examining the extent to which this approximation scales will provide valuable insight into the interpretation of existing and future results using this approximation, independent of whether the underlying assumptions are correct.

We constrain our derivation to the behavior of the LZ approximation for patterns of movement only, and do not explicitly consider parameters such as location dwell time. That is, our analysis is most suited to datasets concerned with trips or trajectories, and will not necessarily apply to datasets which capture prolonged periods of rest. The derivation problem then becomes examining how LZ compression functions for a set of paths.

The most fundamental assumption required for this examination is the definition of a path. We define a human mobility path as a series of piecewise linear two dimensional segments, navigated at a constant velocity. We assume that these paths are executed over a discretized space, as is common in the literature. For convenience, authors of [9, 10, 26] have used non-uniform Voronoi decompositions of the space, as these decompositions flow naturally from the cell tower or WiFi router locations. However, these datasets are characterized by irregular boundaries and variable cell sizes, greatly complicating mathematical derivation of scaling properties. Instead, for tractability, we have chosen a regular grid approximation, which is more appropriately used when discretizing higher fidelity tracked datasets obtained through GPS trackers or smartphone locations [4, 5, 15, 27]. Finally, we assume that paths are sampled regularly in time, again consistent with GPS tracking, rather than the stochastic data arrival associated with cellular call records. Because we assume that we are starting with a high-fidelity source like GPS traces, interpolation of locations between timesteps is not required.

As an agent traverses the discretized space, their locationing system will emit symbols (represented as letters in examples for convenience) corresponding to the label of the grid cells at their measured locations, creating a single dimensional string representing their trajectory through the two dimensional space. Because we assume a piecewise linear path through regular grids, sampled at regular intervals, we can begin to analyze how traversing these grids would appear. For a path parallel to either axis of the grid, the agent will emit a sequence of symbols characterized by repetition of the current grid cell. For constant velocity paths through multiple grid cells, this will lead to a uniform repetition of symbols, based on agent speed and cell size (e.g., ‘AAAABBBBCCCCDDDD’ for one speed and ‘AABBCCDDEEFFGGHH’ for an agent traveling twice as fast). However, if the path is not parallel to the grid cells’ axes, then the agent may clip edges of cell (e.g. ‘AAAAABCCCCC’) changing the string and the entropy rate. As defining all possible arbitrary paths through cells is not mathematically tractable, we assume that agent must traverse the entire cell. This is the strongest assumption that we make, and the most likely to fail when applied to empirical data. This assumptions has the additional impact of forcing paths to be bin-sized aligned; individual line segments must have a length that is an integer multiple of the bin size. Finally, we assume that each line segment traces a unique path through space, and crosses no other segment. While on the surface this seems like a limiting assumption, made to facilitate derivation, we mean to eliminate strongly repeating trajectories, like orbits, which would significantly depress the entropy rate as calculated from the LZ approximation. We expect that crossing but non-overlapping paths, as reported in works such as [11], would have entropy rate approximations close to the unique path case, because while individual symbols might repeat, we would not expect to observe the repetition blocks of multiple symbols.

We limit the analysis to a sampling regime that will return sensible answers. Specifically, we consider regimes for bin width (resolution) and sample period in which scaling is meaningful.

Our assumptions can be summarized as:

1. *Path*: we assume that the path can be sufficiently well approximated as a series of line segments.
2. *Velocity*: we assume a non-zero constant velocity *v* for each line segment *dv*_*i*_/*dt* = 0.
3. *Accuracy*: we assume that a given location measurement offers perfect accuracy, but relax this assumption in additional analysis.
4. *Measurement Density*: we assume that measurements are made with sufficiently high resolution devices so as to support a spatial decomposition into square bins of characteristic length *W* and a regular temporal sampling of period *T*, with no need for interpolation.
5. *Connectedness*: we assume that agents traverse the square bin or block in a classic four-connected manner, that is that participants only move in the cardinal directions though a block and traverse the entirety of the block, implying that the time to traverse a block is always *W*/*v*.
6. *Scale*: we consider a mesoscopic sampling regime with the following characteristics:
  - *Spatial*: the bin size is no bigger than the extent of the smallest line segment in the path.
  - *Temporal*: no cells crossed by the path are skipped due to undersampling: *T* ≤ *W*/*v*.
7. *Independence*: we assume that each segment traces a unique and independent path from all previous segments. This assumption is necessary for tractability, but eliminates repetition (and, therefore, reductions in entropy rate) at an inter-path segment level. Repetition would decrease entropy rate, so we expect that this assumption pushes our derivation towards an upper bound.
8. *Termination*: we assume that each sequence of location symbols terminates with a unique symbol.

In the subsequent sections, we derive scaling behavior from the process of Lempel-Ziv compression, under the above assumptions. For readability, derivations are summarized in the main text. For detailed step-by-step derivations, please refer to S1 Appendix.

### Single Segment Derivation

We begin by considering a single line segment of length *x* traversed at constant velocity *v* parallel to one grid axis, then extend this to multiple non-overlapping line segments. The path requires $t=\frac{x}{v}$ time to traverse. Given our assumptions, the traversal of each grid cell will require at least one sampling period *T* and possibly more, resulting in one of more instances of each cell-symbol being emitted as the agent crosses the cell. Because the agent traverses each cell in its entirety, and in a four-connected manner, it takes the same amount of time to cross each cell. The results is a series of repeated symbols representing each of the cells that the segment passes through, where the number of repeats per cell is given by $L_{b}=\frac{W}{vT}$ and the total length of the string is $L=\frac{x}{vT}$.

From [9, 22, 24], the LZ-derived entropy rate of a string *S* of length *L* is given by

$$\begin{array}{c} {\left(\frac{1}{L}\sum_{i=0}^{L-1}\Lambda_{i}\right)}^{-1}\ln L \end{array}$$ (1)

as *L* → ∞, where *i* is the index of a character in the string (with the first character being at *i* = 0), and Λ_*i*_ is the length of the minimum substring beginning at *i* such that this substring has not previously been observed in the prefix of *S* terminating at position *i*, and *L* is the length of the string.

When scaling the spatial and temporal resolution for simplicity, we consider inter-sample periods given by *T* = *T*_0_2^−*m*^(*m* ≥ 0), and bin sizes as *W* = *W*_0_2^*n*^(*n* ≥ 0), where *W*_0_ and *T*_0_ are governed by our assumptions bounding the bin size and sampling rate.

The values *T*_0_ and *W*_0_ are not necessarily fixed constants, but instead vary with the parameters and the choice of *v*, *x* (for *W*_0_) and *T*. Practically, there are bounds for each, given the method of localization employed, but in our formulation, *W* and *T* are parameters to some degree controlled by the experimenter, while *x* and *v* are properties of the observed agents.

#### Structure of the Sampled Sequence

Both the temporal inter-sampling rate *T* and the spatial scale *W* affect the structure of the sampled sequence. The sequence has a total length of $L=\frac{x}{vT}$ symbols, but is composed of $\frac{x}{W}$ blocks each consisting of $L_{b}=\frac{W}{vT}$ uniform repeating symbols. The number of symbols per block is an interaction between *W*, *T*, and *v*. Larger blocks take longer to traverse, leading to more repeated symbols. For *W* = *x*, the sampled string consists of a single, homogeneous, block of *L* symbols. For our lower bound of *W* = *vT*, this sampled sequence of length *L* consists of $\frac{L}{L_{b}}$ blocks, each consisting of a single unique symbol.

Because we assume non-overlapping paths, the binned values associated with different blocks are distinct. Because the sampled values within a given block are homogeneous, and because the sample value within the block is unique, the values of Λ_*i*_ all follow a regular pattern, *which depends only on the index within the block, and not on the index within the sampled string as a whole*. That is, we will have $\frac{L}{L_{b}}$ unique symbols and blocks, with each symbol repeating *L*_*b*_ times within its block. Thus, $\Lambda_{i}=\Lambda_{i\;\mathrm{mod}\;2^{n}}$, given the structure of our downsampling.

We can thus decompose

$$\begin{array}{c} \frac{1}{L}\sum_{i=0}^{L-1}\Lambda_{i}=\frac{1}{L}\sum_{b=1}^{\frac{x}{vT2^{n}}}\sum_{j=0}^{2^{n}-1}\Lambda_{j} \end{array}$$ (2)

The terms in the outer sum (over *b*) correspond to the number of blocks, which is also the number of unique symbols $\frac{x}{L_{b}}$. The index terms in the inner sum (over *j*) correspond to the number of repetitions in a block of length *L*_*b*_ = 2^*n*^. To derive this sum, we consider two distinct cases: the positions in the first half of the block, and those in the latter half of the block. The pattern for the Λ_*j*_ in the first half of the block is a simple rising sequence. Regardless of the block, the first sample in the block (i.e., *j* = 0) is a unique character not previously seen in the string, and thus ∀_*j* = 0_Λ_*j*_ = 1. Similarly, for all blocks of length of at least 2, the second sample in the block concatenated with its following symbol (in this or the next block) has not previously been seen in the string, and thus ∀_*j* = 1_Λ_*j*_ = 2. Using similar reasoning, the lambda values continue to rise within the block up to the index of $j=\frac{2^{n}}{2}$. Thus $\forall_{j\leq \frac{2^{n}}{2}}\Lambda_{j}=j+1$. That is, for indices up to the halfway point through the string, the substring starting at that point and including *j* additional subsequent characters (and thus of length *j* + 1) consists purely of repetitions of the same character associated with this block, of successively larger lengths, and has not previously been seen. We consider now the cases of the Λ_*j*_ in the second half of the block, noting the assumption above of a unique terminating symbol following characters in the final block. For characters at indices just beyond the midpoint of their block (i.e., $j=\frac{2^{n}}{2}=2^{n-1}$), there is a minimum unique string consisting of the character at that point, $\frac{2^{n}}{2}-1=2^{n-1}-1$ additional identical characters beyond that point lying within the same block, and then (additionally) the first character of the next block, thus yielding a unique total string length starting at position *j* of 2^*n*−1^ + 1 = *j* + 1, as given by the formula above. For the indices in the following 2^*n*−1^ − 1 positions of the string (i.e., for 2^*n*−1^ < *j* ≤ 2^*n*^ − 1), we are dealing with a strictly decreasing integer sequence, terminating in 2. This reflects the fact that for index *j*, the uniform symbol prefixes beginning at index point *j* have all previously been seen within this block, and the smallest unique string consists of the prefix beginning at the current point (index *j*), proceeding through the end of the block, and including one character beyond the end of that block (which has not yet been previously encountered within the string). For a character at position *j* (zero-based) within the block, this yields a string length of (2^*n*^ − *j*) + 1. Thus, we have ∀_*j* > 2^*n*−1^_ Λ_*j*_ = (2^*n*^ − *j*) + 1. To summarize, Λ_*j*_ will be an arithmetic sequence, starting at 1, until just beyond the midpoint is reached; and then decreasing until the final value of 2 (e.g., $1,2,\ldots ,\frac{L_{b}}{2},\frac{L_{b}}{2}+1,\frac{L_{b}}{2},\frac{L_{b}}{2}-1,\ldots ,2$).

Given this per-block total, and that there are $\frac{x}{vT2^{n}}$ blocks, we have:

$$\begin{array}{c} \begin{array}{c} \sum_{j=0}^{2^{n}-1}\Lambda_{j}=\sum_{j=0}^{\frac{2^{n}}{2}}\left(j+1\right)+\sum_{k=0}^{\frac{2^{n}}{2}-1}\left(k+1\right)=\frac{2^{2n}}{4}+2^{n} \end{array} \end{array}$$

Having a closed form expression for Λ_*j*_ and the equivalence in Eq 2, we can now derive an expression for Λ_*i*_.

$$\begin{array}{c} \begin{array}{cc} \frac{1}{L}\sum_{i=0}^{L-1}\Lambda_{i} & =\frac{vT}{x}\frac{x}{vT2^{n}}\left(\frac{2^{2n}}{4}+2^{n}\right)=\left(2^{n-2}+1\right) \end{array} \end{array}$$

Substituting Λ_*i*_ into the equation for LZ compression-based entropy rate Eq (1), the estimated entropy rate of the string is:

$$\begin{array}{c} \begin{array}{cc} H(W,T) & ={\left(2^{n-2}+1\right)}^{-1}\ln\frac{x}{vT}\\ & =\frac{\ln\frac{x}{vT}}{\left(2^{n-2}+1\right)} \end{array} \end{array}$$

Because the number of symbols is related to the width of the cell and sampling rate, and as we have assumed the minimum width *W*_0_ = *vT* to ensure at least one sample per cell

$$\begin{array}{c} \begin{array}{c} H\left(W,T\right)=\frac{4W_{0}\ln\frac{x}{vT}}{\left(W+4W_{0}\right)} \end{array} \end{array}$$

and, therefore,

$$\begin{array}{c} H\left(W,T\right)=\frac{4\ln\frac{x}{vT}}{\frac{W}{vT}+4} \end{array}$$ (3)

Where *x* and *v* are independent properties of the path in question, and *W* and *T* are parameters that are intrinsic to the methods and apparatus of a particular experiment. That a scaling law exists containing only four terms, two controlled by the experimenter, and two determined by the path, is one of the key findings of this work.

While choice of units will affect the size of the *x*, *v*, *T* and *W*_0_ terms, we note that the governing terms $\frac{x}{vT}$ and $\frac{W}{vT}$ are distinguished by being of unit dimension; thus *the entropy rate expression is also of unit dimension, and invariant to unit change*. The first of these expressions is the total length of the sampled string; the latter is the number of samples required to cross a bin. This result suggests that for a single line segment, the entropy rate of strings sampled at different resolutions according to bin widths *W* and temporal inter-sample spacing of *T* should scale proportional to $O(\frac{4\ln\frac{x}{vT}}{\frac{W}{vT}+4})$.

Somewhat counter-intuitively, the entropy rate for a sequence of non-overlapping line segments of total length *x*, which are traversed in four-connected manner, is identical to the single line segment derivation above. Consider two cases: a single line segment of length *x*, and a snaking series of line segments also collectively of length *x*, which are selected in four-connected manner, but randomly picking a non-overlapping direction at every bin. The single segment linear path induces a string containing $\frac{L}{L_{b}}$ unique symbols, each repeating *L*_*b*_ times, as described above, and is, therefore, described by Eq (3). The snaking path induces a string with exactly the same structure. Each transit of a bin produces *L*_*b*_ symbols. At the end of each bin transit, a new batch of *L*_*b*_ symbols begins, starting with a never before seen character. At the end of the path, in accordance with our assumptions, a unique symbol is emitted. This applies to any mixture of line segment lengths traversed at constant velocity, as long as they are multiples of *W*, and do not overlap. Any set of paths that generate a repeating structure like the structure for a single line segment will exhibit entropy scaling behavior described by Eq (3). Intuitively, the straight line trajectory should have a lower entropy rate than the snaking trajectory because the trajectory can be described by a simple mathematical function. However, the entropy rate of the sequence is evaluated independently of the rule used to generate it. This apparent incongruence between the apparent and actual entropy rates for trajectories is subtle, and outside the scope of this work. However, a further investigation into the role of context into human mobility entropy rate estimation, along the lines of [24], appears warranted.

This formulation extends to any number of dimensions as long as the decomposition of that space is a hypercube, and transiting of the hypercube happens hyperface to hyperface along equidistant paths across the hypercube, which is essentially the higher-dimensional generalization of the four-connected path we have assumed. Because the compression—and, therefore, the entropy rate calculation—happens only on the trajectory, which is a single dimensional manifold, as long as the structure of the symbols generated by the trajectory remains the same, the above analysis will hold, and the scaling law will apply. In the case of higher dimensional spaces, *W* is the single dimensional edge length of the hypercube, and *v* is the velocity through the hypercubes. Because opposite faces of a hypercube will be *W* distance apart, by definition, the straight line trajectory through a hyperspace will have the same symbol structure, and, therefore, the same entropy rate scaling behavior as above. Because there also must exist a path of distance *W* between adjacent faces of the hyperplane, the non-overlapping path argument above also applies. Therefore, Eq (3) holds, in general for spaces of arbitrary dimension, decomposed as hypercubes, for non-overlapping paths.

The scaling law exhibits some degree of upper-boundedness against some, but not all, of the assumptions. In particular, paths characterized by repetition will decrease the overall entropy rate by introducing inter-block repetition, that LZ will detect and compress. Violations of the scale assumptions will also decrease entropy, as bin sizes larger than the smallest line segment will cause line segment concatenation with a cell, and, therefore, longer repeating blocks. Similarly, skipping cells due to undersampling will not increase the entropy, as a maximal condition of each symbol in the string being new and unique will already have been reached. However, the addition of noise can disrupt the sequences described here, potentially increasing entropy rate, as expected for additive noise processes. Allowing non-four-connected paths could also increase the entropy in some cases, particularly as cell size increases and clipping becomes more likely, although whether the entropy rate increases or decreases is dependant on the interaction of path and spatial discretization.

#### Scaling Law Behavior

When proposing scaling laws, it is often useful to examine their limiting behavior. The proposed law is well behaved in the limits for the experimenter controlled parameters. As *T* tends to zero, while the length of the string rises, each bin will also be sampled by an ever larger number of repetitions and the entropy rate goes to zero. By contrast, the limit of *H*(*W*, *T*) as *T* → ∞ is negative infinity. However, this bound does not make sense semantically, because it represents the entropy rate of mobility patterns which are never sampled, which violates our assumption about sampling. As *W* approaches zero, entropy rate tends towards a maximum value $\ln\frac{x}{vT}$, which represents the log of the number of symbols sampled, or the entropy rate of a series of distinct symbols of the given length. As *W* → ∞, entropy rate approaches zero, which is sensible, as the entire string would consist of a repetition of the same location symbol.

The proposed law is also well behaved in the path description parameters. As *v* → 0, *H*(*W*, *T*) also goes to zero, as we have a path composed of a single repeating symbol. As *v* → ∞, (putting aside relativistic effects), the entropy rate goes to negative infinity, which, as in the case of *T*, corresponds to a path that is never sampled, and violates our assumptions about sampling. At a minimum, *L* must be at least one, or there is no string, and LZ will return the compression of a single symbol, likely a poor approximation of the entropy rate. As the string becomes infinitely long, with infinite no. of distinct blocks, the entropy rate approaches infinity, as would be appropriate.

A natural question is whether the scaling law has any maxima or minima with respect to *W* or *T*, as this would imply sampling regimes which might be considered optimal. This behavior can be investigated using the partial derivatives. The partial derivative of *H*(*W*, *T*) with respect to *W* is

$$\begin{array}{c} \frac{\partial H}{\partial W}=-\frac{\frac{4}{vt}ln\left(\frac{x}{vT}\right)}{{\left(\frac{W}{vT}+4\right)}^{2}} \end{array}$$ (4)

The derivative does not have a root with respect to *W*, so there are no minima or maxima along the *W* axis for the scaling relationship, implying that no sampling dimension is preferred. Examining the partial derivative of the entropy rate scaling with respect to *T* yields

$$\begin{array}{c} \frac{\partial H}{\partial T}=\frac{4vW+16vT-4vWln(\frac{x}{vT})}{{\left(4Tv+w\right)}^{2}} \end{array}$$ (5)

which has a sequence of roots for a given (*v*, *W*, *x*) at

$$\begin{array}{c} T=\frac{W}{4v}W\left(\frac{4x}{eW}\right) \end{array}$$ (6)

Where *e* is the natural basis and W is the Lambert W function, which is not solvable analytically, but is readily approximated numerically. This function is defined for *W* > 0 and *v* > 0, which is strictly true in our formulation, as *W* is a distance, and *v* is a ratio of distance and time. This implies that for certain values of (*x*, *v*, *W*), there exists a sampling rate corresponding to maximum entropy rate. Sampling beyond this rate will lead to repetition, decreasing the entropy rate. Sampling below this rate will result in removing information, also lowering the entropy rate. This finding is a central outcome of the scaling law, as it implies that there exists an optimal temporal sampling regime for a given spatial resolution and mobility pattern.

### Entropy Rate of Paths with Mixtures of Velocities

While the previous section derived the scaling behavior of the entropy rate of a non-overlapping piecewise linear path, this analysis is unnecessarily limiting for practical application. We seek here to derive an entropy rate for a sequence of non-overlapping line segments traversed with varying velocity. Considering non-overlapping paths as before, Eq (3) provides a starting point to examine how entropy rate might sum for non-overlapping paths of straight line segments through space.

We begin by noting that changes in speed undertaken between two samples occuring within the same spatial bin are not observable, being below the spatial sampling rate. The number of symbols emitted when transiting the cell is proportional to the time it takes to cross the cell, divided by the sampling rate. The time taken to cross the cell can be trivially represented as the width of the cell divided by the average speed within the cell, from the definition of average speed ($\bar{v_{c}}=\frac{W}{T})$. Given that speed changes within a cell are averaged by the emission of symbols, we need only concern the derivation with inter-cell velocity variability.

Given the same linear four-connected path, covering a distance *x*, consider the case where a fraction *α* is made at velocity *βv*, and fraction (1 − *α*) is made at velocity *γv*, yielding a time-averaged velocity of

$$\begin{array}{c} \bar{v}=\frac{v}{\left(\frac{\alpha }{\beta }+\frac{\left(1-\alpha \right)}{\gamma }\right)}. \end{array}$$

The string length is

$$\begin{array}{c} L^{\prime }=\frac{\alpha x}{\beta vT}+\frac{(1-\alpha )x}{\gamma vT}=\frac{x}{\bar{v}T} \end{array}$$ (7)

The total entropy rate is then (step-by-step derivation is provided in S1 Appendix):

$$\begin{array}{c} \begin{array}{cc} & {\left(\frac{1}{L^{\prime }}\sum_{i=0}^{L^{\prime }-1}\Lambda_{i}\right)}^{-1}\ln L^{\prime }\\ & ={\left(\frac{1}{L^{\prime }}\left(\sum_{b=1}^{\frac{\alpha x}{\beta vT2^{n}}}\left(\frac{2^{2n}}{4}+2^{n}\right)+\sum_{b=1}^{\frac{(1-\alpha )x}{\gamma vT2^{n}}}\left(\frac{2^{2n}}{4}+2^{n}\right)\right)\right)}^{-1}\ln L^{\prime } \end{array} \end{array}$$

and therefore

$$\begin{array}{c} H\left(W,T\right)=\frac{4\ln\frac{x}{\bar{v}T}}{\frac{W}{\bar{v}T}+4} \end{array}$$ (8)

which is the same expression as in Eq (3), but including time averaged rather than constant velocity. This derivation is generally valid, subject to bounds on the velocity which maintain that at least one symbol per cell must be recorded, and no cells can be skipped by changing velocity.

### Impact of Spatial Uncertainty

As most entropy rate calculations of interest will be performed on empirical data, it is important to consider the impact of measurement noise on scaling behavior. If measurement noise dominates, then the scaling behavior described here is of limited utility. However, if the measurement noise has well-behaved statistical properties, it may be possible to derive an expected entropy rate considering these impacts. We seek here to consider the effects of spatial noise on the entropy rate estimates, as we expect timing estimates to be much finer grained than human motion. We assume a GPS-like positioning system, with position error estimates that are normally distributed around the true value *μ* with standard deviation *σ*, employing the classic zero mean Gaussian noise model. The probability that a given measurement (a sample from that distribution) lies *further* than distance *d* from the mean is given by $1-erf(\frac{d}{\sigma \sqrt{2}})$.

Now consider taking a measurement at the center point of a generic square bin of physical width *W*. The probability, *p*, of a measurement lying outside the distance to the boundary $\left(\frac{W}{2}\right)$—and, thus, returning an erroneous spatial bin, and associated symbol—is given by Eq (9), where draws from this distribution are considered independent.

$$\begin{array}{c} p=1-erf\left(\frac{W}{2\sqrt{2}\sigma }\right) \end{array}$$ (9)

By incorporating the above noise model, and applying a number of further assumptions, the entropy rate can be approximated as (step by step derivation is provided in S1 Appendix):

$$\begin{array}{c} H\left(W,T\right)=\frac{\ln\frac{x}{vT}}{\frac{1}{p}+\frac{1}{pL_{b}}\left(1+2\left(\frac{\left(1-p\right)\left({\left(1-p\right)}^{\frac{L_{b}}{2}}-1\right)}{p}\right)-{\left(1-p\right)}^{\frac{L_{b}}{2}}\right)} \end{array}$$ (10)

Recall that $L=\frac{x}{vT}$ and $L_{b}=\frac{W}{vT}$, where the total path length is *x*, physical bin width is *W*, the velocity is *v*, and inter-sampling period is *T*. We can further expand Eq (10) by substituting $\frac{W}{vT}$ for *L*_*b*_, and Eq (9) for *p*. If the agent travels distance *x* with a mixture of velocities, *v* in Eq (10) gets substituted by the time-averaged velocity $\bar{v}$.

Erroneous symbols generated through noise processes may come from a bin traversed earlier in the trajectory, a bin that will be traversed later in the trajectory, or from a bin that will not be encountered by the trajectory. While the occurrence of an erroneous reading in either of the first two categories will yield repetitions (thus, preventing the relevant substrings from being entirely unique), an occurrence of the latter will not. Specifically, we believe that it is considerably more likely that the formula in Eq (10) will underestimate the entropy rate in practice, as large enough noise to be effective will disrupt the repetition of symbols, and, therefore, increase entropy rate. However, it is possible to imagine pathological behavior where noise would, for the entire duration it takes to traverse a bin width *W* at *v*, perturb the measurement in the direction of the next bin on the trajectory, returning a double length sequence of symbols and thus decreasing the entropy rate. However, for a symmetric error distribution like a Gaussian, we anticipate that this behavior should be rare.

**Fig 1:**
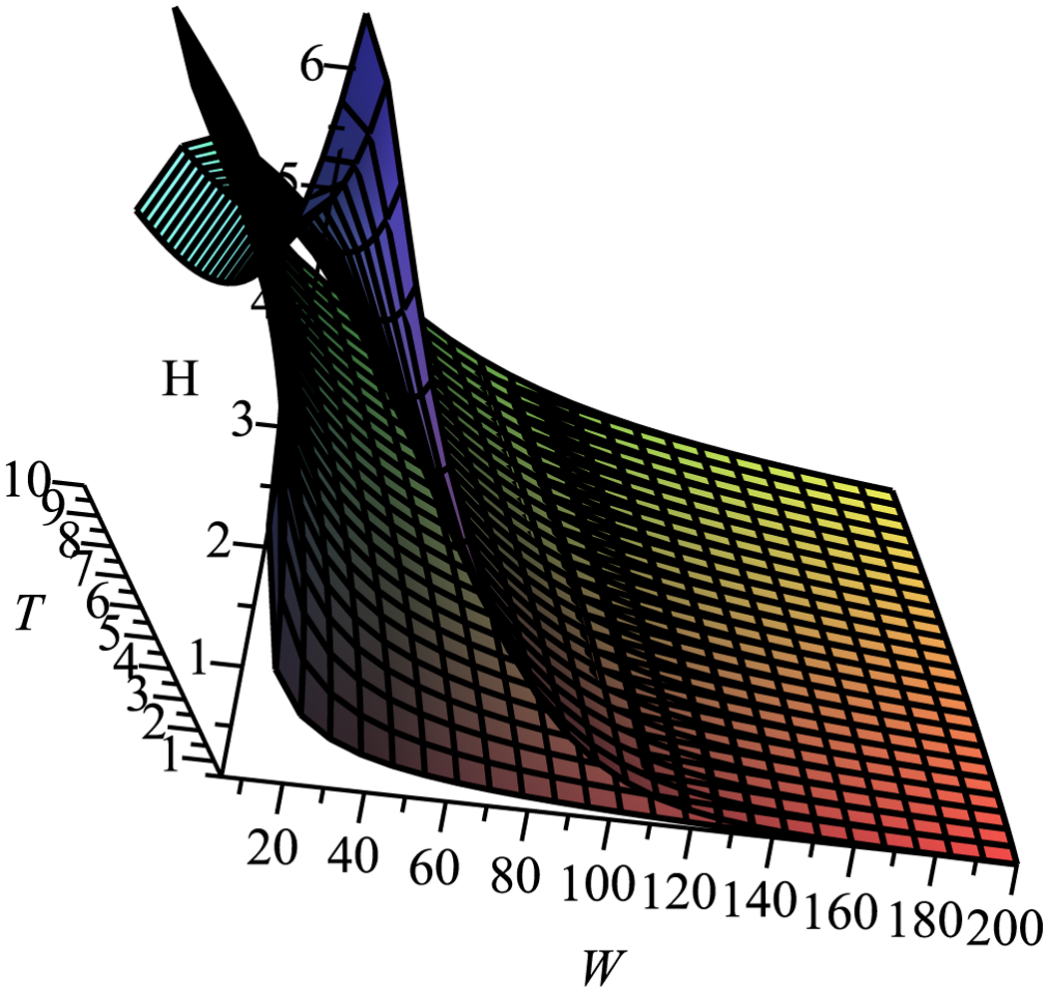
Entropy Rate measures with (generally top) and without noise (generally bottom).

Fig 1 compares the entropy rate measures with (generally top) and without (generally bottom) noise for 5 < = *W* < = 200, 0.5 < = *T* < = 10, $\bar{v}=1$, and *x* = 1000. Absent noise, the entropy rate is generally lower over wide ranges of medium and large spatial scales and sampling periods when compared with the estimate of entropy rate with noise. However, at small physical scales and longer sampling periods, the entropy rate absent noise can lead to sequences of entirely unique symbols, whereas there is some repetition in the presence of noise—and, therefore, somewhat lower entropy rate. Assuming a standard deviation of 30*m* for GPS, these two entropy rate estimates exhibit a high degree of disparity, particularly for physical scales of around 40−80*m*. By contrast, the entropy rate estimates with and without noise approach each other asymptotically as the spatial aggregation scale increases, as expected.

## Methods

To provide a semi-empirical validation for the model, we compared the results of the theoretical model with the results from two widely employed and stylized simulated models of human mobility. A single agent traversed a simulated field with a constant speed (*v*) while following the employed motion models, and agent locations on the grid were recorded according to the spatial and temporal sampling rates. The maximum and minimum sampling periods were set to 512*s* and 1*s*, respectively. We collected 64 samples for *max*(*T*) = *T*_0_ = 512 *s*; therefore, making the number of samples 64 × 2^*m*^ for *T* = *T*_0_2^−*m*^. To collect 64 samples at *T*_0_ = 512*s*, the agent in the theoretical model had to traverse 64*vT*_0_ = 65536*m*. For other models where the agent moved in a square field, we set the diagonal length of the field to 64*vT*_0_ to make their comparison with the theoretical model sensible. The minimum value of *W* for a combination of *v* and *T* is *vT*, and the maximum value of *W* is 64*vT*_0_. Each models was applied with and without power law distributed dwelling at nodes, and (for each such variant) with and without additive noise. The two empirical motion models are:

- *Random Waypoint Motion Model*: in this model, 100 unique waypoints were drawn uniformly from the field described above. The waypoints described a fully connected graph; that is, the agent could go from a waypoint to any other waypoint. This allows crossing paths, which we assumed absent in the theoretical derivation for simplicity. Transitions from one waypoint to another were drawn uniformly. However, because waypoints were drawn uniformly, the probability of repeated path sequences is low. We investigated transitions with and without dwell time. For transitions with dwell time, dwell time was drawn from a power law distribution with the exponent of −1.8 and maximum dwell time was set to 17 hours, consistent with [10].
- *Power Law-based Motion Model*: in this model, the agent selected an angular direction uniformly from a set $\left\{5k^{{}^{\circ}}:k\in N^{+}\;\text{and}\;5\leq 5K\leq 355\right\}$, and drew the distance for the next step from a power law distribution, which is typically observed in empirical datasets (e.g., [10]). Draws were constrained to ensure that the agent remained in the field. The distance was limited to 0.8 times the characteristic length of the field. Movement directions were resampled until a destination inside the field was generated. In these experiments, −1.55 was chosen as the power law exponent, consistent with reported empirical findings [10]. For the dwell time variant, we employed the same distribution as for the Random Waypoint model.

We also considered an additive measurement noise model. Each of the above scenarios was run once without any additive noise and once for the noise model. Simple zero mean Gaussian additive measrement noise model was considered, consistent with simple noise models of GPS location measurements. Noise was added to the signal after the agent moved but before simulated measurement took place. A moderate (*σ* = 10*m*) noise level was selected consistent with commodity GPS systems. A theoretical entropy rate was calculated from Eq (3), and compared to the empirical measurement calculated according to Eq (1).

Several aspects of these simulated motion models depart from the assumptions made when deriving our scaling law. First, each model permits crossing paths, leading to repeated symbols, although are unlikely to produce cyclic paths. Second, we have included variants which include measurement noise and dwelling, neither of which are explicitly accounted for in Eq (3). Third, the models can lead to clipping effects explicitly ruled out when deriving Eq 3.

Given that the paths were generated in simulation, we have precise control over the sampling rates, bin widths, path length and agent velocity and can, therefore, explicitly calculate the scaling law, and compare them against the Lempel-Ziv derived entropy rates from the trajectory records. Employing bin widths of $W=W_{0}2^{n}=vT2^{n}$, we can simplify Eq (3) into Eq (11).

$$\begin{array}{c} H\left(W,T\right)=\frac{4ln(L)}{2^{n}+4} \end{array}$$ (11)

We use the coefficient of determination (*R*^2^ metric) to understand how well the theoretical curves fit with those from the empirical simulation models, including the model that applies Eq (1) to the sequences of the theoretical model. The definition of *R*^2^ is given in Eq (12), where *f*_1_, *f*_2_, …, *f*_*n*_ are the predicted values for *y*_1_, *y*_2_, …, *y*_*n*_. *R*^2^ values were calculated in R software environment.

$$\begin{array}{c} R^{2}=1-\frac{\sum_{i}^{n}{\left(y_{i}-f_{i}\right)}^{2}}{\sum_{i}^{n}{\left(y_{i}-\frac{1}{n}\sum_{i}^{n}y_{i}\right)}^{2}} \end{array}$$ (12)

We ran the simulations on a Linux-based computing cluster with 96 computational nodes, each having 2 x eight-core Intel E5-2650L (1.8GHz) or Intel E5-2640L (2.0 GHz) Xeon Processors, and 32GB RAM. Jobs were submitted to the cluster through the Torque scheduler. Refer to S1 Data for the relevant data and code required to generate the data.

## Results

We seek to determine how well the scaling law behaves when compared to paths absent non-Gaussian measurement noise, participant non-compliance and other effects that may be present in empirical data, which might obfuscate the underlying behavior and make comparisons more difficult. Some of the simulated systems here are noise free, but do allow for repeating symbols and cell clipping. Analyzing the behavior of these simulated systems against the theoretical scaling model could provide insight into the impact of breaking these key assumptions on the proposed scaling law’s predictions.

**Fig 2:**
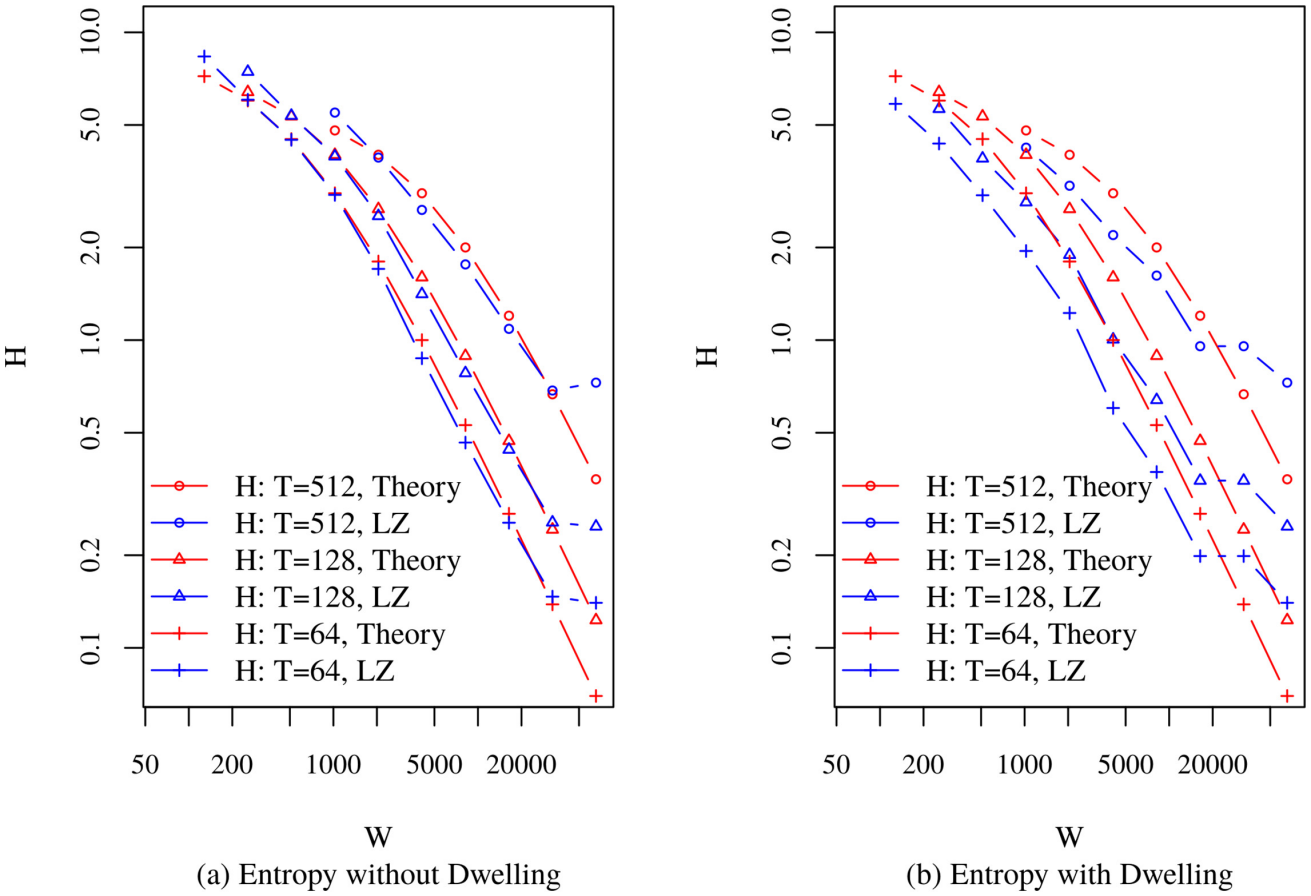
Theoretical Model Generated Sequence Entropy Rate Vs. LZ Entropy Rate of Sequence Obtained from Power Law Models.

Fig 2 presents the comparison between the theoretical model and power law-based models with and without dwelling, and with no added measurement noise in the sequences. In the model without dwelling, the scaling law provides exceptional agreement with the simulation. At very large *W*, the empirical entropy rate exceed the theoretical, as clipping effects begin to dominate. As the bin width increases, more repetitions occur in the string. Therefore, entropy rate goes down. The theoretical model considers regular patterns of string. However, because of stochastic nature of empirical strings, the effect of large bin width may be less dominant in lowering the entropy rate than is the case for the theoretical model. This is why the entropy rate of the empirical models in Fig 2 for large *W* exceeds that of the theoretical model. As an example, consider two 64-character strings from the alphabet {‘0’, ‘1’}, which are expressed, using regular expression, as /0{32}1{32}/ and /1{3}0{31}1{30}/. Here, the second string has a higher entropy rate. The first string has the structure assumed by the theoretical model, while the second indicates a clipped trajectory. The latter may appear as the representation of a trip, at a large bin width, which is derived from power law-based trip segment lengths and dwell times.

**Fig 3:**
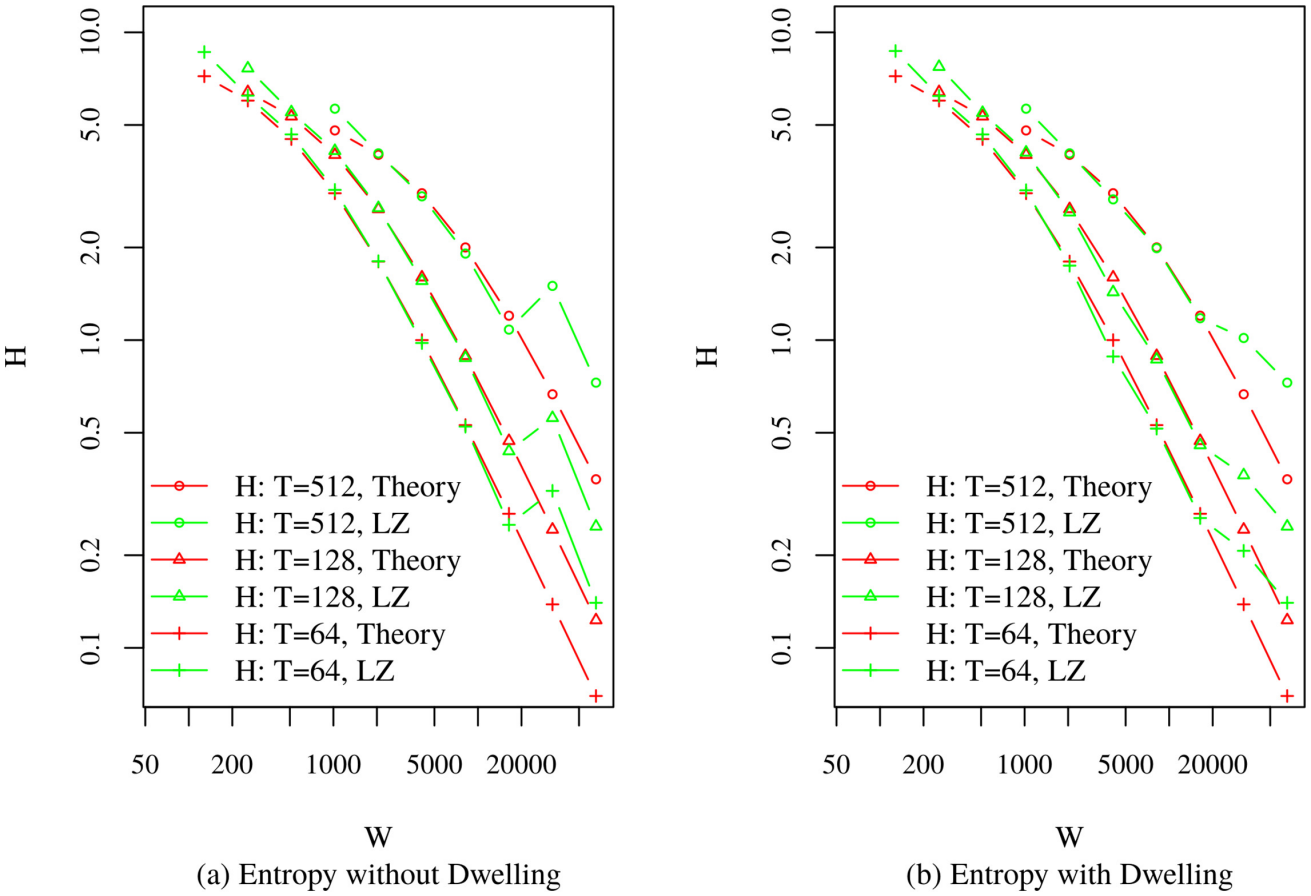
Theoretical Model Generated Sequence Entropy Rate Vs. LZ Entropy Rate of Sequence Obtained from Random Waypoint Models.

Fig 3 presents the comparisons between the theoretical model and the noise-free random waypoint-based models with and without dwelling. Similar to the power law based empirical model, entropy rates at large bin widths exceed those of the theoretical model. However, the effect of dwelling is less pronounced than power law-based models, because fewer constraints were placed on the trip length in the random waypoint model. The trip segments, therefore, were longer and fewer trip segments (2 to 5 segments as compared to 186 to 292 for the power law model in the conducted experiments) were required to obtain the desired numbers of location samples. This resulted in fewer dwell occurrences in the random waypoint model than their power law counterparts. The theoretical model shows admirable agreement for the entropy rate scaling behavior for both synthetic mobility models. Deviation from theoretical behavior is apparent for very small and very large values of *W*.

**Fig 4:**
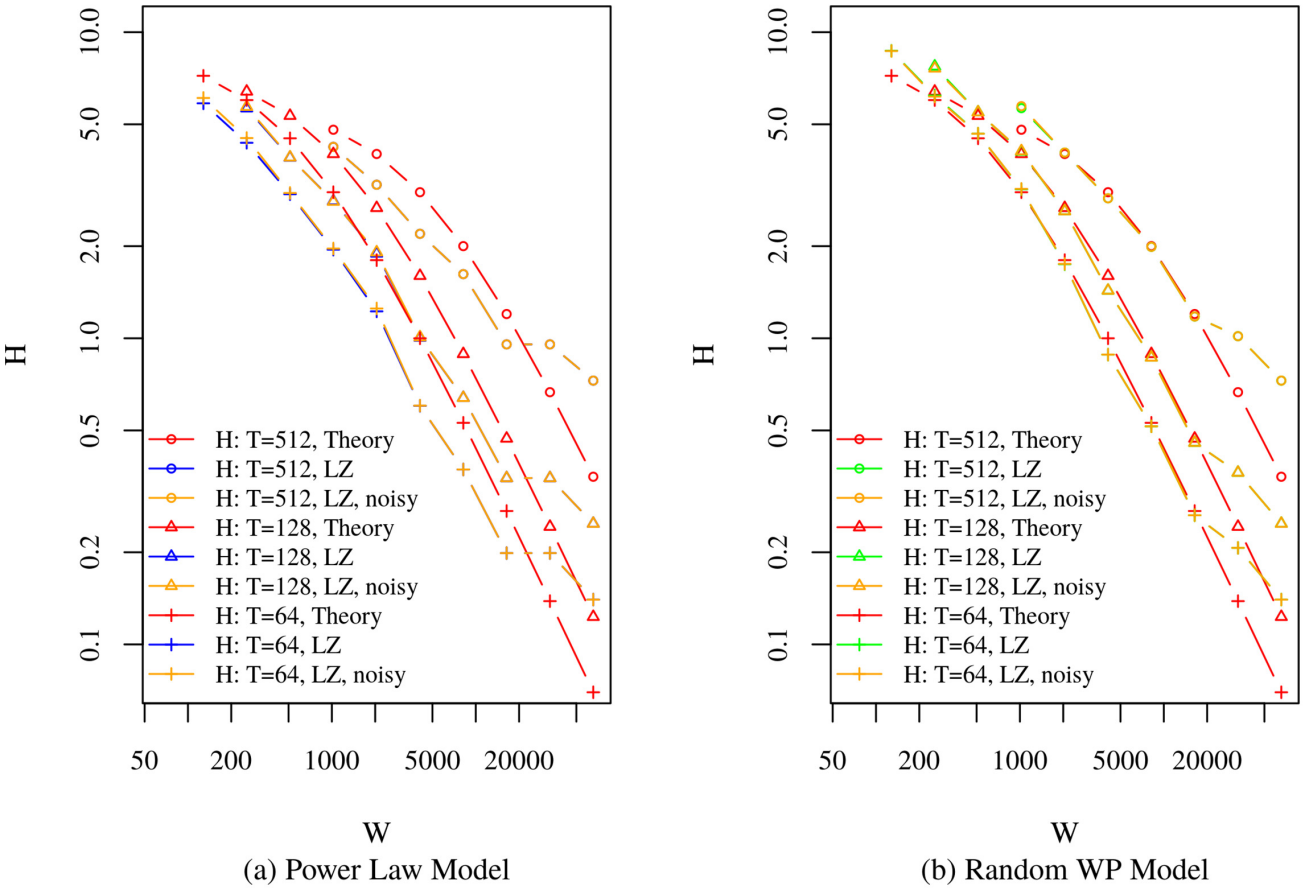
Theoretical Model Generated Sequence Entropy Rate Vs. LZ Entropy Rate of Power Law and Random Waypoint Models with and without Noise, and with Dwelling.

To show the effects of added measurement noise to the power law and random waypoint based models on entropy rate, Fig 4 presents the entropies of the sequences obtained from these models, with dwelling enabled, alongside the entropies of their noisy versions for *σ* = 10*m*, a value typical for consumer GPS systems. Fig 4 shows that the introduced zero mean Gaussian noise does not significantly alter the entropy rate, particularly as grid size increases. The probability that a given measurement falls outside the current grid cell, given the accuracy of GPS systems, is small for the sizes of cells considered. Smaller cells would be more susceptible to noise deviations, and might show greater impact on entropy rate, but that impact would be predominantly sensor noise and not the phenomenon of interest. While compensating for noise using more complex models such as Eq (10) may be possible, a simpler solution in some circumstances would be to use bin sizes larger than the expected error, but that still capture the phenomenon of interest.

**Fig 5:**
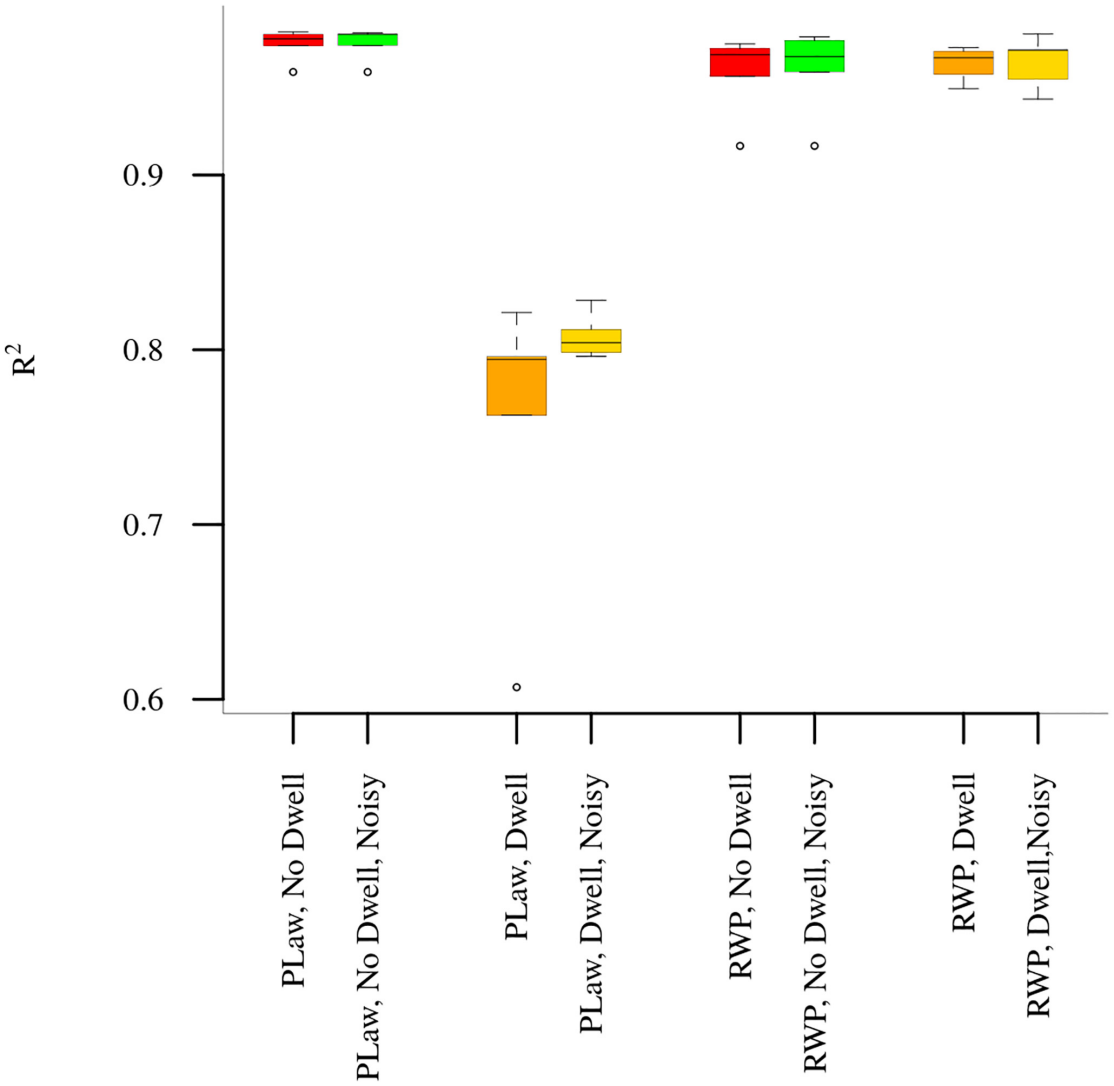
Fitness of Theoretical Curves to Simulation Models.

Fig 5 compares the curves generated by the theoretical and simulation models. For each simulation model, we compare the curves, relating entropy *H* to *W* for different values of *T*, with the corresponding curves of the theoretical model. Each boxplot in Fig 5 is generated with the *R*^2^ values of fitting the theoretical curves to the curves of the simulation models over all *T*. All but the power law with dwelling model show exceptional fit quality (in excess of 0.9), and even the poorer fitting models have an *R*^2^ of about 0.8. The shortcomings of the *R*^2^ metric on non-linear models notwithstanding, these results provide us with additional confidence in the fit quality visually evident in the previous figures.

### Explanation of Results

The theoretical model provides a surprising degree of agreement with the synthetic mobility models, suggesting that the mechanics of compression have a great deal to do with the scaling behavior reported in the literature. Our derivation indicated that, subject to our assumptions, the scaling model should form an upper bound on the entropy rate, as any deviations from a unique straight line path would reduce repetition in the string, and, therefore, increase the entropy rate. However, when the theory deviates from the prediction, it almost always underestimates the entropy rate calculated from Lemple-Ziv compression. This is primarily due to violations of two of our assumptions, made to make the mathematics tractable.

First, while we assumed a unique termination character during our derivation, we did not supply a unique termination character at the end of strings built from the simulation. This has the counterintuitive result of increasing the estimated entropy rate. Consider a sequence of four symbols. If all symbols are the same, $\sum_{i=0}^{L-1}\Lambda_{i}=8$ under our assumption, compared to $\sum_{i=0}^{L-1}\Lambda_{i}=3$ according to Eq (1). Therefore, theoretical entropy rate drops faster than the LZ-entropy for larger *W*.

Second, we assumed that the agent traversed the entirety of each block that it encountered; however, this is not necessarily the case in practice. For example, a path which traverses cell A, clips cell B and traverses cell C could have a corresponding location string of ‘AAAAAAAACCBBBBBB’, whereas the theory implicitly assumes that the path must be ‘AAAAAAAABBBBBBBB’. While this assumption was reasonable at small *W*, at larger scales, real paths are less likely to transit in a four connected manner. This effect also demonstrates that there are representational effects in the compression calculation. With grid and travel path at arbitrary relative orientations, paths which clip the edge of a cell are possible, and increasingly likely with increasing cell size, increasing the entropy rate at larger scales beyond the theoretical prediction.

However, despite these shortcomings, the predicted values showed excellent agreement with the empirical values computed from LZ compression on simulated paths. These results are encouraging for extending our model to incorporate real empirical data, which is confounded by missing data, varying sample sizes and non-Gaussian noise processes. This model should provide a firm theoretical basis for continuing work to address the more difficult situations encountered in real data.

## Discussion

In this paper, we have described a methodology for estimating the differences in predicted entropy rates over different spatial and temporal scales, with and without Gaussian noise, grounded in the theoretical behaviour of the Lempel-Ziv compression algorithm typically used to the calculate mobility entropy rate. We have demonstrated that scaling behaviour is to be expected and is inversely proportional to the spatial scale, and proportionate to the logarithm of the sampling rate. From these derivations, we were able to demonstrate that there is a predicted sampling rate of maximal entropy rate, which can be calculated using the Lambert W function. This theoretical model was validated against models of simulated movement, and found to provide excellent fits for stylized results, but with declining impact at very large or small spatial scales where our assumptions begin to break down. These results are important for a number of reasons.

First, we establish a strong theoretical foundation for mobility entropy rate scaling behavior observed and reported by a number of other authors [24, 26]. Based on an analysis of the behavior of Lempel-Ziv compression on the kinds of strings created by agents moving through space, we were able to demonstrate that the mobility entropy rate scaling behavior could be described with only four terms: the length of the path, the average velocity of the agent, the width of the spatial bin, and the period of the sampling rate. Because the scaling law encodes both parameters related to agent motion (*x*, *v*) and experimental design (*W*, *T*), we can conclude that the scaling depends both on agent behavior and the mathematical realization of that path. This finding is important, as it indicates that the scaling behavior encodes the mobile agent’s behavior, and is not purely an artifact of mathematics, and, therefore, is itself a potentially useful metric. This finding also opens a clear opportunity to separate the two components of entropy rate scaling, providing the ability to isolate the behavioral fingerprint represented in the data.

Second, the scaling law is general, subject to the assumptions. Because the trajectory compressed using Lempel-Ziv itself is a single dimensional manifold, as long as the space decomposition and path definition is analogous to the four-connected path described in the assumptions, the scaling law is valid. Similarly, because LZ compression does not distinguish between symbols, only symbol order, any non-overlapping path that crosses the entirety of a cell along only cardinal directions is also valid. We note that while describing the trajectories of people was our primary motivation, this derivation applies to the trajectory of any agent moving through space, subject to our assumptions.

Third, the structure of the equation indicates that the differences matter. As shown in the results and in previous works [24, 26], changing the scale of measurement can have a significant impact on the resulting entropy rate calculation. Directly comparing mobility entropy rates from experiments with differing spatial and temporal resolutions is not meaningful. Estimates of entropy rate at a common spatio-temporal resolution, either using the upper bound estimate here, or through an empirical estimate, would be required. This outcome is particularly important for spatial scale, as it implies that the results for studies with heterogeneous cell sizes may be confounded by scaling effects, particularly if the frequency of visits to cells of different sizes is significantly different for different participants.

Finally, the scaling law has a maximum value with respect to *T*, implying that there is a preferred sampling rate for a given spatial and velocity profile. This is an obvious point to use as a common comparator between datasets. Datasets with similar entropy rate maxima will likely have more similar scaling properties than those that do not. This property is also potentially useful for researchers designing data collection studies, as they could use anticipated average velocity, trip length and spatial bin size to identify a preferred sampling period *T*.

### Limitations and Future Work

The primary limitation in this work is the set of assumptions which made the theoretical analysis tractable. By assuming that the agent was always in motion, and that the path contained no repetitions, and through use of a simple noise model, we have constrained the generalizability of the findings. However, the model matched well against simulated systems, and is relatively straightforward to calculate. The primary goal of any future work should be to extend our results to encapsulate a more broadly representative model of human mobility and noise processes. The second major limitation of our assumptions was that the discretization of space was based on equally dimensioned square grid cells. While this is a reasonable assumption, in practice, researchers have employed cellular tower records to provide the discretization of space (e.g. [10]), leading to a distribution of cell sizes based on the Voronoi diagram of the cell towers’ spatial configuration. The irregularity of the cell tower configuration could potentially exacerbate cell clipping effects, and make the entropy rate dependent on the path the agent takes though the cell. A more sophisticated analysis treating both cell shape and path orientation as independent random variables might address these issues; however, that analysis requires a substantial additional body of research. Similarly, time scales from call records are not constant and depend on individual calling patterns. Extending our work so that spatial resolution and sampling rate can also be represented as random variables would be an important step forward. Finally, we validated our scaling law against simulated mobility models. The model provided surprisingly good fits given the strength of the assumptions, and the fact that both simulated systems violated those assumptions. However, the stylized mobility models employed, while popular, have been shown to be imperfect representations of human mobility [11, 28]. It is a priority to validate the scaling law against actual mobility data.

#### Concluding Remarks

The findings presented here provide a theoretical explanation for the scaling behavior observed in calculations of mobility entropy rate from strings of locations using Lempel-Ziv compression. These results, while based on stylized assumptions, provided a useful approximation of scaling behavior for a wide variety of simulated paths, knowing only the average velocity, even under simulated sensor noise. The theory and simulated results provided close agreement for a wide range of spatial and temporal sampling scales, only breaking down at relatively large (corresponding to long repetitions of single symbols) or very small (corresponding to strings of unique symbols) spatial scales, indicating that our assumptions are plausibly valid. The entropy rate scaling formulation has a maximum at a particular sampling frequency, implying that optimal sampling regimes for given trajectories should exist and are in principle approximatable. This work is an important step in transforming mobility entropy rate from a scientific curiosity into a reliable workhorse of modern mobility and spatial behavior studies. By extending this work to emprical data and less stylized mobility assumptions, a scale-free mobility entropy rate formulation may be derived.

## Supporting Information

S1 Appendix Detailed Scaling Law Derivation. (PDF)

S1 Data Relevant data and program code to generate the data. (ZIP)
