# Supplementary material for: A Theoretical Basis for Entropy-Scaling Effects in Human Mobility Patterns
Source: PLoS One. 2016 Aug 29;11(8):e0161630. doi: 10.1371/journal.pone.0161630 (PMC5003381; doi:10.1371/journal.pone.0161630)
Supplement: S1 Appendix — (PDF) [file pone.0161630.s001.pdf]

S1 Appendix. Detailed Scaling Law Derivation.

Supporting Material for

A Theoretical Basis for Entropy-scaling Effects in Human Mobility Patterns

Nathaniel D. Osgood, Tuhin Paul, Kevin G. Stanley, and Weicheng Qian

Entropy Rate Estimate

Given the joint pmf distribution of the realizations $\{X_t\}_{\tau+1}^{\tau+n}$ of a stochastic process, The *block entropy* is defined as:

$$H_{\tau,n} = - \sum_{x_{\tau+1}, x_{\tau+2}, \ldots, x_{\tau+n}} p(x_{\tau+1}, x_{\tau+2}, \ldots, x_{\tau+n}) \log p(x_{\tau+1}, x_{\tau+2}, \ldots, x_{\tau+n}) \tag{1}$$

Because the blocks can be of arbitrary length, concepts like differential entropy or entropy rate become more relevant than Shannon's entropy. The differential entropies for the process used in (1) are:

$$h_{\tau,n} = H_{\tau,n} - H_{\tau,n-1} \tag{2}$$

Differential entropy measures the new information introduced by the nth outcome, having known the preceding $(n-1)$ outcomes. If we consider a stationary process, we can ignore τ to make the mathematical expressions easier to follow. Differential entropy can also be expressed as:

$$\begin{aligned} h_n &= - \sum_{x_1, x_2, \ldots, x_n} p(x_1, x_2, \ldots, x_n) \log p(x_n | x_1, x_2, \ldots, x_{n-1}) \\ &= H(X_n | X_{n-1}, X_{n-2}, \ldots, X_1) \end{aligned} \tag{3}$$

To consider all correlations and constraints in the realizations of the process, the average amount of information per symbol (entropy rate) is defined in the limiting case as follows:

$$h = \lim_{n \to \infty} h_n \tag{4}$$

In (4), h_n converges monotonically to h from above for a stationary stochastic process. For a stationary stochastic process, $p(x_1, x_2, \ldots, x_n)$ can be computed from a finite sequence of length N, where $N > n$ [1]. However, with increasing n, the number of combinations of $(x_1, x_2, \ldots, x_n)$ in (3) increases exponentially and so does the minimum value of N to faithfully compute probabilities $p(x_1, x_2, \ldots, x_n)$. This renders (3) unrealistic as a practical means for computing the metric. Therefore, researchers have proposed alternative methods to approximate the entropy rate from finite length symbol sequences. The estimator of (5), which is based on the LZ compression algorithm,

converges fast, and has been widely used to approximate the entropy rate of human mobility traces [2, 3, 4]. The LZ compression algorithm parses a symbol sequence $\{x_k\}_{k=1}^{n}$ into words such that the next word to parse is the shortest sequence unseen in the past. The words are encoded as (j, c) pairs, where c is the last character of the next word to encode and j is the codeword index of the word that corresponds to all but the last character of the next word to encode. The first symbol constitutes the first word and is encoded as $(0, x_1)$.

In this supplementary document, we supply the line-by-line derivations to accompany the main paper. The derivations here are subject to the same assumptions posited in the main document. For readability of the detailed derivations, many of the key equations and arguments have been reproduced here. Our goal is to determine the spatiotemporal scaling behavior for Lempel-Ziv compression, according to the following equation.

$$H = \left(\frac{1}{L} \sum_{i=0}^{L-1} \Lambda_i \right)^{-1} \ln L \tag{5}$$

The entropy rate of a string S of length L is given by (5) as $L \to \infty$, where i is the index of a character in the string (with the first character being at $i = 0$), and Λ_i is the length of the minimum substring beginning at i such that this substring has not previously been observed in the prefix of S terminating at position i. Now consider the sequences of characters of that string S resulting from sampling the agent's location along a 1D trajectory at different levels of spatial and temporal resolutions.

Ranges of Spatial and Temporal Resolution

For simplicity, we consider spatial and temporal sampling rates which scale by powers of two. For each spatial scale, we consider only temporal inter-sampling period regimes in which at least one sample will be measured within the time for the agent to traverse the distance x; that is, in general, $T \leq T_0 = \frac{x}{v}$, which is the upper limit (T_0) of inter-sampling time, as shown in (6).

$$T_0 = \frac{x}{v} \tag{6}$$

and, for the general case within this range,

$$T = \frac{T_0}{2^m} \qquad \text{where } m \in \mathbb{N}^0 \tag{7}$$

The spatial bins are bounded by the following assumptions.

Upper bound: $W = x$**.** At and above this level of spatial scale, all samples from the path are mapped to the same bin.

Lower bound: $W = W_0 = vT$**.** Below this spatial scale, we begin missing transited cells due to undersampling, and all sampled blocks remain unique.

$$W_0 = vT = v\left(\frac{T_0}{2^m}\right) \tag{8}$$

and, for the general case within this range,

$$W = W_0 2^n \qquad \text{where } n \in \mathbb{N}^0 \tag{9}$$

Structure of the Sampled Sequence

Both the temporal inter-sampling rate T and the spatial scale W affect the structure of the sampled sequence. At the most fundamental level, the length of the sampled sequence representing the trajectory varies with temporal inter-sampling period T, being given by $L = \frac{x}{vT}$ characters. Moreover, the internal structure of the sequence will differ across both temporal resolutions T and spatial resolutions W. An important insight is that this sequence itself consists of a series of uniformly sized *blocks*, each composed uniformly of a repeated occurrence of a single unique character.

Both the spatial scale and the temporal sampling rate strongly impact this structure. For $W = x$ (i.e., a bin width equal to the total path length), the sampled string consists of a single and homogeneous block of length $L = \frac{x}{vT}$ characters. For

the lower mesoscopic bound of the bin size $W = vT$, this sampled sequence of length L consists of $\frac{x}{vT}$ blocks, each of length 1 and consisting of a unique sampled character (reflecting the fact that at the maximal resolution, the successive samples all fall into distinct bins). In general, for a specific temporal scale (associated with inter-sampling time T) and the resolution associated with bin width $W = W_0 2^n$, the sample string of length L will consist of $\frac{x}{W}$ successive blocks, each of length $\frac{W}{vT}$, and each consisting purely of repetitions of one sampled value - the bin into which all of the sampled locations within that block fall; and each such block will be associated with a unique such sampled value. As alluded to above, the minimal spatial scale yielding a change in entropy rate is $W = vT$; below that level of scale W, entropy rate will remain invariant, as the length of the sequence depends only on temporal scale T, and the sampled values will remain unique and equal in number. For a given level of temporal scale T, we thus can specify $W_0 = vT$, and consider spatial scaling at successive binary powers n of that minimum scale. Thus, for a given n, $W = W_0 2^n$, and we will have N_b blocks as shown in (10), each of length L_b characters, as shown in (11).

$$N_b = \frac{x}{W} = \frac{x}{W_0 2^n} = \frac{x}{vT2^n} \tag{10}$$

$$L_b = \frac{W}{vT} = \frac{W_0 2^n}{vT} = \frac{vT2^n}{vT} = 2^n \tag{11}$$

Because of our assumptions of $1D$ trajectories and (to this point) constant speed, the binned values associated with different blocks are distinct and the sampled values within a given block are homogeneous. Therefore, the values of Λ_i all follow a regular pattern, *which depends only on the index within the block, and not on the index within the sampled string as a whole.* Thus, $\Lambda_i = \Lambda_{(i \mod 2^n)}$. We can thus decompose the sum over the entire string ($\sum_{i=0}^{L-1} \Lambda_i$) into nested sums over blocks b and indicies i within each such block, as in (12):

$$\begin{aligned}\frac{1}{L}\sum_{i=0}^{L-1}\Lambda_i &= \frac{1}{L}\sum_{i=0}^{L-1}\Lambda_{(i \mod 2^n)} \\ &= \frac{1}{L}\sum_{b=1}^{\frac{x}{vT2^n}}\sum_{j=0}^{2^n-1}\Lambda_j\end{aligned} \tag{12}$$

We now consider the total of the Λ_j values across a block, $\sum_{j=0}^{2^n-1}\Lambda_j$. Because the value of Λ_j depends only on the location of the block (i.e., $\Lambda_i = \Lambda_{(i \mod 2^n)}$), this sum over the Λ_j within a block is identical for different blocks. To derive this sum, we consider two distinct cases – the positions in the first half of the block, and those in the latter half of the block.

The pattern for the Λ_j in the first half of the block is a simple rising sequence. Regardless of the block, the first sample in the block (i.e., $j = i \mod 2^n = 0$) is a unique character not previously seen in the string, and thus $\forall_{j=0}\Lambda_j = 1$. Similarly, for all blocks of length of at least 2, the second sample in the block concatenated with its following symbol (in this or the next block) has not previously been seen in the string, and thus $\forall_{j=(i \mod 2^n)=1}\Lambda_j = 2$. Using similar reasoning, the lambda values continue to rise within the block up to the index of $j = 2^n/2$ (zero-based). Thus $\forall_{j=(i \mod 2^n)\leq\frac{2^n}{2}}\Lambda_j = j+1$. That is, for indices up to the halfway point through the string, the substring starting at that point and including j additional subsequent characters (and thus of length $j+1$) consists purely of repetitions of the same character associated with this block, of successively larger lengths, and has not previously been seen.

We consider now the cases of the Λ_j in the second half of the block. Before discussing the handling of this case, we note that after the final block of the entire string of length L, we assume either a unique terminating character, or the starting character of the initial block, which has never previously been encountered following characters in the final block. We now turn to discuss the characters in the latter half of blocks in general. For characters at indices just beyond the midpoint of their block (i.e., $j = i \mod 2^n = \frac{2^n}{2} = 2^{n-1}$), there is a minimum unique string consisting of the character at that point, $2^{n-1}-1$ additional identical characters beyond that point lying within the same block, and then (additionally) the first character of the next block,

thus yielding a unique total string length starting at position j of $2^{n-1}+1=j+1$, as given by the formula above. For the indices in the following $2^{n-1}-1$ positions of the string (i.e., for $2^{n-1}<j\le 2^n-1$), because the uniform symbol prefixes beginning at index point j have all previously been seen within this block, the smallest unique string consists of the prefix beginning at the current point (index j), proceeding through the end of the block, and including one character beyond the end of that block (which has not yet been previously encountered within the string). For a character at position j (zero-based) within the block, this yields a string length of $(2^n-j)+1$. Thus, we have $\forall_{j=(i \mod 2^n)>2^{n-1}} \Lambda_j = (2^n-j)+1$. Therefore, we can decompose the sum of Λ_j values in a block as follows:

$$
\begin{aligned}
\sum_{j=0}^{L_b-1}\Lambda_j &= \sum_{j=0}^{\frac{L_b}{2}}(j+1) + \sum_{j=\frac{L_b}{2}+1}^{L_b-1}(2^n-j+1) \\
&= \sum_{j=1}^{\frac{L_b}{2}+1} j + \sum_{2^n-j'=\frac{L_b}{2}+1}^{2^n-j'=L_b-1}\left(j'+1\right) \quad \text{, where } j'=2^n-j \\
&= \sum_{j=1}^{\frac{L_b}{2}+1} j + \sum_{L_b-j'=\frac{L_b}{2}+1}^{L_b-j'=L_b-1}\left(j'+1\right) \quad \text{, given that } L_b=2^n \text{ from (11)} \\
&= \sum_{j=1}^{\frac{L_b}{2}+1} j + \sum_{j'=\frac{L_b}{2}-1}^{j'=1}\left(j'+1\right) \\
&= \sum_{j=1}^{\frac{L_b}{2}+1} j + \sum_{j'=1}^{\frac{L_b}{2}-1}\left(j'+1\right) \\
&= \sum_{j=1}^{\frac{L_b}{2}} j + \frac{L_b}{2} + 1 + \sum_{j'=1}^{\frac{L_b}{2}-1} j' + \frac{L_b}{2} - 1 \\
&= \sum_{j=1}^{\frac{L_b}{2}} j + \frac{L_b}{2} + \sum_{j'=1}^{\frac{L_b}{2}} j' \\
&= 2\sum_{j=1}^{\frac{L_b}{2}} j + \frac{L_b}{2}
\end{aligned}
\tag{13}
$$

From the above (13), we have the sum of the Λ_j across a single block, that is $\sum_{j=0}^{2^n-1}\Lambda_j = \sum_{j=1}^{\frac{L_b}{2}}\Lambda_j$, as given by $2\sum_{j=1}^{\frac{L_b}{2}} j + \frac{L_b}{2}$. Now recognizing that $\sum_{k=1}^{c} k = \frac{c(c+1)}{2}$,

the above can be further reduced as follows:

$$
\begin{aligned}
\sum_{j=0}^{2^n-1} \Lambda_j &= 2\sum_{j=1}^{\frac{L_b}{2}} j + \frac{L_b}{2} \\
&= 2\frac{(\frac{L_b}{2}(\frac{L_b}{2}+1)}{2} + \frac{L_b}{2} \\
&= \frac{L_b}{2}(\frac{L_b}{2}+1) + \frac{L_b}{2} \\
&= (\frac{L_b}{2})^2 + \frac{L_b}{2} + \frac{L_b}{2} \\
&= \frac{(L_b)^2}{4} + L_b \\
&= \frac{(2^n)^2}{4} + 2^n \quad \text{, given that } L_b = 2^n \text{ from (11)} \\
&= \frac{2^{2n}}{4} + 2^n
\end{aligned}
\tag{14}
$$

Given the formula in formula 14 above for the sum of the Λ_j across a single block, we turn our attention now to their sum across all blocks $\frac{1}{L}\sum_{i=0}^{L-1}\Lambda_i$, as is considered in (12). By applying $L = \frac{x}{vT}$ and (14) into (12), we have:

$$
\begin{aligned}
\frac{1}{L}\sum_{i=0}^{L-1}\Lambda_i &= \frac{1}{\frac{x}{vT}}\sum_{b=1}^{\frac{x}{vT2^n}}\left(\frac{2^{2n}}{4}+2^n\right) \\
&= \frac{vT}{x}\frac{x}{vT2^n}\left(\frac{2^{2n}}{4}+2^n\right) = \\
&= \frac{1}{2^n}\left(\frac{2^{2n}}{4}+2^n\right) = \\
&= \left(\frac{2^n}{4}+1\right) = \left(2^{n-2}+1\right)
\end{aligned}
\tag{15}
$$

Recalling from (5) that $H = \left(\frac{1}{L}\sum_{i=0}^{L-1}\Lambda_i\right)^{-1}\ln L$, and recalling that $L = \frac{x}{vT}$, and (from (9)) that $W = W_0 2^n$, the formula for the entropy rate of the string can be simplified below in (16) to:

$$
\begin{aligned}
H = \left(\frac{1}{L}\sum_{i=0}^{L-1}\Lambda_i\right)^{-1}\ln L &= \left(2^{n-2}+1\right)^{-1}\ln\frac{x}{vT} = \frac{\ln\frac{x}{vT}}{(2^{n-2}+1)} \\
&= \frac{\ln\frac{x}{vT}}{\left(\frac{\left(\frac{W}{W_0}\right)}{4}+1\right)} = \frac{4W_0\ln\frac{x}{vT}}{(W+4W_0)}
\end{aligned}
\tag{16}
$$

Recall that the basal (minimum meaningful) spatial scale W_0 varies with the temporal resolution, reflecting the fact that more finely temporally sampled paths can benefit from additional precision on the spatial scale (and thus a smaller bin size at which the sample begins to return unique values). Specifically, recall from (9) that $W_0 = vT$. Thus, for the joint scaling relation, we have

$$\frac{4W_0 \ln \frac{x}{vT}}{(W + 4W_0)} = \frac{4vT \ln \frac{x}{vT}}{(W + 4vT)} = \frac{4 \ln \frac{x}{vT}}{\left(\frac{W}{vT} + 4\right)} \tag{17}$$

While choice of units will affect the size of the x, v, T and W_0 terms, we note that the governing terms $\frac{x}{vT}$ and $\frac{W}{vT}$ are distinguished by being of unit dimension; thus *the entropy rate expression is also of unit dimension, and thus invariant to unit change.* The first of these expressions is the total length of the sampled string; the latter is the number of samples required to cross the bin size. This result suggests that given a continuous, one-dimensional trajectory, the entropy rate of strings sampled at different resolutions according to bin widths W and temporal inter-sample spacing of T should scale as $O\left(\frac{4 \ln \frac{x}{vT}}{\frac{W}{vT}+4}\right)$.

Entropy of Paths with Mixtures of Velocities

We now consider traversing the same distance x, but where a fraction of the distance α is made at velocity βv, and fraction $(1-\alpha)$ is made at velocity γv. For this case, the total elapsed trip time is $\frac{\alpha x}{\beta v} + \frac{(1-\alpha)x}{\gamma v} = \frac{x}{v}\left(\frac{\alpha}{\beta} + \frac{(1-\alpha)}{\gamma}\right)$. This yields a time-averaged velocity of $\bar{v}$, shown in (18).

$$\bar{v} = \frac{x}{\frac{x}{v}\left(\frac{\alpha}{\beta} + \frac{(1-\alpha)}{\gamma}\right)} = \frac{v}{\left(\frac{\alpha}{\beta} + \frac{(1-\alpha)}{\gamma}\right)} \tag{18}$$

The corresponding string length, L', is given in (19).

$$
\begin{aligned}
L' &= \frac{x}{\bar{v}T} \\
&= \frac{x}{vT}\left(\frac{\alpha}{\beta} + \frac{(1-\alpha)}{\gamma}\right) \\
&= \frac{\alpha x}{\beta v T} + \frac{(1-\alpha)x}{\gamma v T}
\end{aligned} \tag{19}
$$

The total entropy rate is then calculated according to (20):

$$
\begin{aligned}
&\left(\frac{1}{L'}\sum_{i=0}^{L'-1}\Lambda_i\right)^{-1}\ln L' = \left(\frac{1}{L'}\sum_{i=0}^{L'-1}\Lambda_i\right)^{-1}\ln\left(L'\right) \\
&= \left(\frac{1}{L'}\left(\sum_{b=1}^{\frac{\alpha x}{\beta v T 2^n}}\left(\frac{2^{2n}}{4}+2^n\right) + \sum_{b=1}^{\frac{(1-\alpha)x}{\gamma v T 2^n}}\left(\frac{2^{2n}}{4}+2^n\right)\right)\right)^{-1}\ln\left(L'\right) \\
&= \left(\frac{1}{\frac{x}{vT}\left(\frac{\alpha}{\beta}+\frac{(1-\alpha)}{\gamma}\right)}\left(\frac{\alpha x}{\beta v T 2^n}\left(\frac{2^{2n}}{4}+2^n\right) + \frac{(1-\alpha)x}{\gamma v T 2^n}\left(\frac{2^{2n}}{4}+2^n\right)\right)\right)^{-1}\ln\left(L'\right) \\
&= \left(\frac{1}{\left(\frac{\alpha}{\beta}+\frac{(1-\alpha)}{\gamma}\right)}\left(\frac{\alpha}{\beta 2^n}\left(\frac{2^{2n}}{4}+2^n\right) + \frac{(1-\alpha)}{\gamma 2^n}\left(\frac{2^{2n}}{4}+2^n\right)\right)\right)^{-1}\ln\left(L'\right) \\
&= \left(\frac{1}{\left(\frac{\alpha}{\beta}+\frac{(1-\alpha)}{\gamma}\right)}\left(\frac{\alpha}{\beta}\left(\frac{2^{n}}{4}+1\right) + \frac{(1-\alpha)}{\gamma}\left(\frac{2^{n}}{4}+1\right)\right)\right)^{-1}\ln\left(L'\right) \\
&= \left(\frac{1}{\left(\frac{\alpha}{\beta}+\frac{(1-\alpha)}{\gamma}\right)}\left(\left(\frac{\alpha}{\beta}+\frac{(1-\alpha)}{\gamma}\right)\left(\frac{2^{n}}{4}+1\right)\right)\right)^{-1}\ln\left(L'\right) \\
&= \left(\frac{1}{\left(\frac{\alpha}{\beta}+\frac{(1-\alpha)}{\gamma}\right)}\left(\left(\frac{\alpha}{\beta}+\frac{(1-\alpha)}{\gamma}\right)\left(\frac{2^{n}}{4}+1\right)\right)\right)^{-1}\ln\left(L'\right) \\
&= \left(\frac{2^n}{4}+1\right)\ln\left(L'\right) = \frac{\ln\left(\frac{x}{\bar{v}T}\right)}{\left(\frac{\frac{W}{\bar{v}T}}{4}+1\right)} \\
&= \left(\frac{\frac{W}{\bar{v}T}}{4}+1\right)^{-1}\ln\left(\frac{x}{\bar{v}T}\right) \\
&= \frac{4\ln\frac{x}{\bar{v}T}}{\frac{W}{\bar{v}T}+4}
\end{aligned} \tag{20}
$$

We emphasize that the above is the same as the formula for the entropy rate $H = \frac{4W_0 \ln \frac{x}{vT}}{(W+4W_0)}$ derived in (16) for the case of a fixed velocity, except that the mean velocity $\bar{v}$ is substituted for originally fixed entropy v. While the analysis above considered

two segments at different velocities, the derivation readily generalizes *mutatis mutandis* to other mixtures of velocities.

Impact of Spatial Uncertainty

It is well recognized that positioning systems such as GPS are associated with noise. We consider here the effects of such spatial noise on the entropy rate estimates. Employing the classic zero mean Gaussian noise model, we assume that GPS positioning is associated with measurements that are normally distributed around the true value μ with standard deviation σ. The probability of a given GPS measurement (a sample from that distribution) lying further than distance y from the mean is given by $1 - erf\left(\frac{y}{\sigma\sqrt{2}}\right)$. Now consider taking a measurement at the center point of a unidimensional bin of physical width W, which is measured in the same unit system as y. The probability of a unidimensional measurement lying outside the distance to the boundary, $\frac{W}{2}$, is given by:

$$p = 1 - erf\left(\frac{\frac{W}{2}}{\sigma\sqrt{2}}\right) = 1 - erf\left(\frac{W}{2\sqrt{2}\sigma}\right) \tag{21}$$

Now consider a sequence of measurements, as considered earlier in this document. In the presence of noise, we can relate the Λ_j values within a block to a *truncated geometric distribution*. A random variable Y following a truncated geometric distribution with probability p of success and up to k tries, where the kth draw is a success if all previous ones fail, has an expected value given in (22). For simplicity and as an approximation, we consider the draw associated with each element of the sum in (22) as independent, and as occurring from the center of the bin.

$$\begin{aligned} E[Y] &= \sum_{i=1}^{k-1} i\left((1-p)^{i-1}p\right) + k\left(1 - \sum_{i=1}^{k-1}\left((1-p)^{i-1}p\right)\right) \\ &= \frac{1-(1-p)(1-p)^{k-1}}{p} \\ &= \frac{1-(1-p)^k}{p} \end{aligned} \tag{22}$$

To compute Λ_j for each index j of samples in a block, we assume that a sample ends the unique sequence starting at j if the sample is erroneously reported to lie outside of the current bin. That is, we consider that if an incorrect value is sampled (i.e., if the positioning system erroneously reports a location outside of the current bin), that it will represent a repetition that terminates any unique sequence. If we consider a draw at a given sample position j, we treat the number of tries to obtain an erroneous value as following a truncated geometric distribution, where the number of tries is bound by the the length of the unique sequence that would start at position j when noise is absent. This maximum value is dictated by the position and the probability of achieving a value from outside of the bin is given by the value p. Therefore, Λ_j can be approximated by the expected value of this truncated geometric distribution. For the case of multiple draws from this distribution, associated with determining Λ_j at position j, we consider the discrepancy in the measurements independent. Adapting the formula in (13) for the probabilistic case, we can decompose the sum of the Λ_j for the block as the below, where the "times to repeat up to m" times are considered to reach the value m if and only if no erroneous reading has occurred, and is otherwise immediately truncated.

$$2\sum_{j=1}^{\frac{L_b}{2}} (\text{times to repeat up to } j \text{ tries}) + \text{times to repeat up to } \frac{L_b}{2} \text{ tries}$$

For our simplified case, we, therefore, approximate a total of the Λ_j across the current block of

$$
\begin{aligned}
&2\sum_{j=1}^{\frac{L_b}{2}} \frac{1-(1-p)^j}{p} + \frac{1-(1-p)^{\frac{L_b}{2}}}{p} \\
&= \frac{1}{p}\left(2\left(\sum_{j=1}^{\frac{L_b}{2}} 1-(1-p)^j\right) + 1-(1-p)^{\frac{L_b}{2}}\right) \\
&= \frac{1}{p}\left(2\left(\frac{L_b}{2} - \sum_{j=1}^{\frac{L_b}{2}}(1-p)^j\right) + 1-(1-p)^{\frac{L_b}{2}}\right) \\
&= \frac{1}{p}\left(2\left(\frac{L_b}{2} - \left(\frac{(1-p)\left((1-p)^{\frac{L_b}{2}}-1\right)}{(1-p)-1}\right)\right) + 1-(1-p)^{\frac{L_b}{2}}\right) \\
&= \frac{1}{p}\left(2\left(\frac{L_b}{2} + \left(\frac{(1-p)\left((1-p)^{\frac{L_b}{2}}-1\right)}{p}\right)\right) + 1-(1-p)^{\frac{L_b}{2}}\right) \\
&= \frac{1}{p}\left(\left(L_b + 2\left(\frac{(1-p)\left((1-p)^{\frac{L_b}{2}}-1\right)}{p}\right)\right) + 1-(1-p)^{\frac{L_b}{2}}\right) \\
&= \frac{1}{p}\left(L_b + 1 + 2\left(\frac{(1-p)\left((1-p)^{\frac{L_b}{2}}-1\right)}{p}\right) - (1-p)^{\frac{L_b}{2}}\right)
\end{aligned} \tag{23}
$$

Now, summing up across the $N_b = \frac{L}{L_b}$ blocks, we have a denominator to (5) of:

$$
\begin{aligned}
\frac{1}{L}\sum_{i=0}^{L-1}\Lambda_i &= \frac{1}{L}\sum_{b=1}^{\frac{L}{L_b}}\left(\frac{1}{p}\left(L_b + 1 + 2\left(\frac{(1-p)\left((1-p)^{\frac{L_b}{2}}-1\right)}{p}\right) - (1-p)^{\frac{L_b}{2}}\right)\right) \\
&= \frac{1}{L}\frac{L}{L_b}\left(\frac{1}{p}\left(L_b + 1 + 2\left(\frac{(1-p)\left((1-p)^{\frac{L_b}{2}}-1\right)}{p}\right) - (1-p)^{\frac{L_b}{2}}\right)\right) \\
&= \frac{1}{pL_b}\left(L_b + 1 + 2\left(\frac{(1-p)\left((1-p)^{\frac{L_b}{2}}-1\right)}{p}\right) - (1-p)^{\frac{L_b}{2}}\right) \\
&= \frac{1}{p} + \frac{1}{pL_b}\left(1 + 2\left(\frac{(1-p)\left((1-p)^{\frac{L_b}{2}}-1\right)}{p}\right) - (1-p)^{\frac{L_b}{2}}\right)
\end{aligned} \tag{24}
$$

By substituting (24) into (5), we can express the entropy rate, in the presence of white noise, as:

$$H = \frac{\ln \frac{x}{vT}}{\frac{1}{p} + \frac{1}{pL_b}\left(1 + 2\left(\frac{(1-p)\left((1-p)^{\frac{L_b}{2}} - 1\right)}{p}\right) - (1-p)^{\frac{L_b}{2}}\right)} \tag{25}$$

Recall that $L = \frac{x}{vT}$ and $L_b = \frac{W}{vT}$, where the total path length is x, physical bin width is W, the velocity is v, and inter-sampling period is T. We can further expand (25) by substituting $\frac{W}{vT}$ for L_b, and (21) for p. If the agent travels distance x with a mixture of velocities, v in (25) is substituted by the time-averaged velocity $\bar{v}$.

References

1. Schürmann T, Grassberger P. Entropy estimation of symbol sequences. Chaos: An Interdisciplinary Journal of Nonlinear Science. 1996;6(3):414–427.

2. Kontoyiannis I, Algoet PH, Suhov YM, Wyner AJ. Nonparametric entropy estimation for stationary processes and random fields, with applications to English text. Information Theory, IEEE Transactions on. 1998;44(3):1319–1327.

3. Rodriguez-Carrion A, Garcia-Rubio C, Campo C, Das SK. Analysis of a fast LZ-based entropy estimator for mobility data. In: Pervasive Computing and Communication Workshops (PerCom Workshops), 2015 IEEE International Conference on. IEEE; 2015. p. 451–456.

4. Song C, Qu Z, Blumm N, Barabási AL. Limits of predictability in human mobility. Science. 2010;327(5968):1018–1021.
